# The safety of telemedicine clinics as an alternative to in-person preoperative assessment for elective laparoscopic cholecystectomy in patients with benign gallbladder disease: a retrospective cohort study

**DOI:** 10.1186/s13037-023-00368-7

**Published:** 2023-08-29

**Authors:** Tomas Urbonas, Adil Siraj Lakha, Emily King, Sophia Pepes, Carlo Ceresa, Venkatesha Udupa, Zahir Soonawalla, Michael A Silva, Alex Gordon-Weeks, Srikanth Reddy

**Affiliations:** Department of hepatobiliary surgery, Churchill Hospital, Oxford University Hospitals NHS Foundation Trust, Oxford, England

**Keywords:** Elective surgery, Day case surgery, Laparoscopic cholecystectomy, Preoperative assessment, Telemedicine, Virtual clinics, Remote healthcare, Telesurgery, General surgery.

## Abstract

**Background:**

The telemedicine clinic for follow up after minor surgical procedures in general surgery is now ubiquitously considered a standard of care. However, this method of consultation is not the mainstay for preoperative assessment and counselling of patients for common surgical procedures such as laparoscopic cholecystectomy. The aim of this study was to evaluate the safety of assessing and counselling patients in the telemedicine clinic without a physical encounter for laparoscopic cholecystectomy.

**Methods:**

We conducted a retrospective analysis of patients who were booked for laparoscopic cholecystectomy for benign gallbladder disease via general surgery telemedicine clinics from March 2020 to November 2021. The primary outcome was the cancellation rate on the day of surgery. The secondary outcomes were complication and readmission rates, with Clavein-Dindo grade III or greater deemed clinically significant. We performed a subgroup analysis on the cases cancelled on the day of surgery in an attempt to identify key reasons for cancellation following virtual clinic assessment.

**Results:**

We identified 206 cases booked for laparoscopic cholecystectomy from telemedicine clinics. 7% of patients had a cancellation on the day of surgery. Only one such cancellation was deemed avoidable as it may have been prevented by a face-to-face assessment. Severe postoperative adverse events (equal to or greater than Clavien-Dindo grade III) were observed in 1% of patients, and required re-intervention. 30-day readmission rate was 11%.

**Conclusions:**

Our series showed that it is safe and feasible to assess and counsel patients for laparoscopic cholecystectomy remotely with a minimal cancellation rate on the day of operation. Further work is needed to understand the effect of remote consultations on patient satisfaction, its environmental impact, and possible benefits to healthcare economics to support its routine use in general surgery.

## Introduction

During the COVID-19 pandemic, the entirety of the National Health System (NHS) was forced to reduce patient contact, impacting the way in which healthcare was provided[1] [2]. One of the major adaptations in outpatient hospital services and general practice settings was the use of telemedicine clinics[3]. The telemedicine clinic in general surgery has become widespread since the pandemic onset and has remained so following relaxation of restrictions on conventional in-person appointments. Prior to the COVID-19 pandemic, telemedicine clinics had been commenced as a seemingly safe way to follow up patients after elective surgical procedures [4] [5] [6] [7]. During the pandemic, general surgery telemedicine clinics were used in part to assess and diagnose patients and consider their suitability for various invasive procedures[8, 9]. Furthermore, these virtual clinics were useful to plan and counsel patients for operations.

Telemedicine clinics throughout the pandemic allowed all patients and particularly those who were vulnerable, to be assessed without putting them at increased risk of contracting COVID-19. Post-pandemic, telemedicine clinic appears to be more accessible than a face-to-face appointment for patients given the busy demands of modern life; they can more easily be taken whilst, for example, at work. This may allow patients who previously may have delayed receiving medical attention to access it more easily. They are also beneficial to healthcare providers as they do not require outpatient facilities which saves cost[10] and allows more patients to be reviewed per clinic which decreases waiting times. Usage of telemedicine clinics is also more environmentally friendly, as they reduce carbon footprint, hospital footfall, and FFP2 mask usage[11] [12] [13].

An In-person clinical examination is traditionally viewed as a crucial component of patient evaluation and treatment planning in surgical practice. Hence, a major concern related to telemedicine clinics is that patients could not be adequately assessed and counselled for the need of an operation as well as their risks of having an invasive procedure. The inability to examine a patient may result in missed crucial information or important clinical factors which could adversely affect patient outcomes. Communication over the telephone may also be poorer between clinician and patient when compared with face-to-face appointments where non-verbal communicative cues may give the clinician important insights into the patients’ understanding and opinions. Despite these concerns, there have been reports of satisfactory telemedicine clinic outcomes for patients and providers in surgical specialities[14] [15] [16] [17] including even paediatric surgery [18]. Utilisation of telemedicine clinics has been shown to improve the outcomes of treatment in conditions such as vascular leg ulcers[19].

One study analysed large-scale trends of telemedicine conversion during the pandemic for surgical visits, and although this study found an increase in telemedicine utilisation, the overall uptake rate of telemedicine visits remained low, suggesting unidentified barriers to widespread telemedicine adoption in surgical care [20].

There is a concern that it is unsafe to assess patients for surgical intervention virtually, which in theory could result in increased complication rates due to inadequate assessment and underestimation of risks although it hasn’t been studied previously. Although the concept of virtual postoperative follow up in surgery has been widely adopted [21, 22], preoperative telemedicine assessment even for common invasive procedures hasn’t been unanimously accepted. The utilisation of such clinics for preoperative assessment of patients for the commonest surgical procedures if done safely could potentially have a massive impact on healthcare practice and economics[10]. It was shown that remote consultations were beneficial for liver transplant patients in terms of saving time and money, being less burdensome, and causing fewer negative impacts on patients’ health[23].

This study aims to assess whether telemedicine can be routinely used for the preoperative assessment of patients undergoing common general surgery procedures, in particular laparoscopic cholecystectomy as one the commonest operations performed in general surgery[24]. Our primary outcome of interest was the proportion of patients who experienced cancellation of surgery on the day of admission for laparoscopic cholecystectomy.

## Methods

### Demographics

The study was conducted in a tertiary centre in the United Kingdom. We conducted a retrospective analysis of laparoscopic cholecystectomies booked via general surgery telemedicine clinics from 23 to 2020 to 30 November 2021. This covers the period between when a lockdown was imposed on United Kingdom residents and the move to ‘Plan B’ with the spread of the Omicron variant in December 2021. All patients aged 16 and older who were assessed, counselled and listed for laparoscopic cholecystectomy for benign gallbladder disease via general surgery telemedicine clinic were included for analysis. We define benign pathology of the gallbladder to include indications such as calculous cholecystitis, biliary colic, gallstone pancreatitis and gallbladder polyps. No malignant (gallbladder cancer) operations were included in our analysis and any patients with gallbladder cancer were excluded from our cohort. Cancellation, 30-day readmission and 30-day complication rates using the Clavein-Dindo classification system [25] were calculated. Clavein-Dindo grade III + complications were deemed clinically significant, and so these have been scrutinised further and presented in the results section below.

### Telemedicine process

All patients were assessed on the phone in terms of their complaints and history of presenting complaints, past medical and surgical history, review of laboratory biochemistry and haematology, and scan results. The only aspect of assessment which wasn’t performed was physical examination due to the nature of virtual clinic. All patients were counselled on the phone and a standardised information leaflet was posted to all patients for completeness of the preoperative counselling process. All patients were also routinely assessed by a specialist nurse in our preoperative assessment clinic shortly prior to surgery. High-risk patients identified during the initial clinic assessment were routinely referred for anaesthetic assessment to pre-emptively identify and evaluate comorbidities which may impact fitness for surgery. The American Society of Anaesthesiologists (ASA) physical status was generated from past medical history of the patient using medical notes [26].

### Data analysis

Cancellations were further analysed on a per-patient basis to understand whether they could have been avoided through physical patient assessment.

Continuous variables were presented as means with standard deviation for normally distributed data and medians with range for non-normally distributed data as determined using the Shapiro–Wilk test. Categorical variables were presented as frequencies and percentages. Statistical analysis was performed using JASP Version 0.16.2.

## Results

Overall, we identified 240 patients who were assessed and booked for surgery after assessment in a telemedicine clinic. 206 required laparoscopic cholecystectomy for benign gallbladder disease and therefore were included into the final analysis. Of all 240 reviewed cases assessed and booked for surgery from telemedicine clinic, 187 (78%) patients had gallstones, 19 (8%) gallbladder polyps, 13 (5.4%) secondary liver tumour (colorectal metastases), 5 (2%) benign liver cyst, 5 (2%) primary pancreatic tumour, 4 (1.7%) primary liver tumour, 1 (0.4%) gallbladder tumour and 6 (2.5%) other pathologies. Of these, only benign gallbladder disease patients were included (gallstones and polyps). The baseline characteristics of the study population are summarized in Table 1.

**Table 1:** Study population (n = 206)

Baseline characteristics		Value	Range/percentage
Total patients		206	
Age (median)		47	16–81
Male, n (%)		56	27%
BMI (median)		29	15.3–56.8
Referred for anaesthetic assessment		17	8%
ASA	1	73	35.5%
	2	108	52.4%
	3	25	12.1%
Days between clinic and surgery (median) n = 206		91	3-383
Planned for day case, n (%) n = 206		192	93%
Cancellation on the day, n (%)		14	7.0%
Stayed as a day case, n (%) N = 192		161	84%
Readmission, n (%) n = 192		21	11%
Complications (Clavien-Dindo) n = 225	1	14	6.8%
	2	21	10.9%
	3a	1	0.5%
	3b	1	0.5%
	4a	0	0%
	4b	0	0%

There were 14 (7.0%) cancellations on the day of the surgery which are summarized in Table 2. 13 cancellations were unavoidable (Table 2) and only one of them was deemed potentially avoidable as the patient was not suitable for a day case procedure due to previous bariatric surgery and high BMI. This cancellation could have been avoided had the patient been assessed face-to-face manner.

**Table 2:** Cancellations

Reason of cancellation	Number	Percentage	Avoidable
Positive covid swab prior to admission	1	0.5%	No
Pyrexia on the day of surgery (suspected Covid infection)	2	1%	No
Worsening renal function (creatinine and eGFR) on the day of surgery	1	0.5%	No
Unavailability of inpatient beds due to covid on the day of surgery	4	2%	No
Uncontrolled hypertension on the day of surgery [27]	1	0.5%	No
Diarrhoea on the day of surgery	1	0.5%	No
High blood glucose on the day of surgery (poorly controlled diabetes)	1	0.5%	No
Anaesthetic staff shortage	1	0.5%	No
Not suitable for day case (cancelled on the day as deemed unsafe to proceed as a day case)	1	0.5%	Yes
Anxiety on the day of surgery	1	0.5%	No
Total	14	7%	

21 patients (11%) of those who had surgery were readmitted within 30 days after discharge. The causes of readmissions are summarised in Table 3. Most of the readmitted patients didn’t require re-intervention and were managed conservatively either with extra analgesia, antiemetics or antibiotics. One patient (0.5%) was readmitted with an infected collection in the gallbladder bed, requiring laparoscopic drainage and wash out of this collection (a Clavien-Dindo IIIb complication). One patient (0.5%) was readmitted with a surgical site infection at the umbilical incision and required incision and drainage under local anaesthetic (a Clavien-Dindo IIIa complication). Therefore, the 30-day reintervention rate in our cohort of patients was 1%.

**Table 3:** Causes of 30-day readmission after surgery

Cause	(n = 192)
Abdominal pain	10 (5.25%)
Nausea and vomiting	2 (1.0%)
Surgical site infection (SSI)	8 (4.25%)
Surgical bed collection	1 (0.5%)
Total	21 (11%)

The American Society of Anaesthesiologists (ASA) physical status was generated from past medical history of the patient using medical notes [26]. In our cohort majority of the patients were ASA 1–2 and only 12.1% were classified as ASA 3 (Table 1).

Overall, cancellation rate on the day of the surgery was low (7.0%) with only one cancellation (1/206 = 0.5%) deemed due to inadequate virtual assessment of the patient. 30-day readmission rate of the entire study population was 11% and significant complication rate (Clavien-Dindo 3a and above) was 1%.

## Discussion

Benign gallbladder disease is a major global health burden. It is estimated that up to 15% of western population develops gallstones with 4% per year becoming symptomatic [28]. Thus, patients with benign gallbladder disease take up a large proportion of outpatient general and benign hepatobiliary outpatient clinics. The feasibility and safety of exclusively virtual assessment and counselling of patients who undergo laparoscopic cholecystectomy have not been widely studied.

The analysis of the outcomes of the patients undergoing laparoscopic cholecystectomy booked and assessed from telemedicine clinic from this study showed that it is feasible and safe to continue its utilisation alongside conventional face-to-face appointments for selected patients. Cancellation, complication and 30-day readmission rates were used as surrogate endpoints of telemedicine clinic safety and feasibility. There were 14 (7.0%) operations cancelled on the day of the surgery, however only one (0.5%) was deemed to be related to inadequate telephone assessment of the patient and therefore potentially avoidable. Crudely, this is a similar proportion when compared with elective day case cancellation rates seen in larger studies published elsewhere [29]. The complication and readmission rates in our cohort of patients is comparable to national rates [30] [31] [32].

Due to consistent overbooking and relatively long waiting times, the desire is to continue telemedicine clinics at least for follow-up patients and newly referred low-risk patients, which could decrease the waiting time for appointments and improve patients’ access to healthcare professionals[33]. Telemedicine clinic may also be advantageous for the patients who are spared the cost and inconvenience of commute[34]. Another potential advantage of virtual clinics is reduced no-show rates which would positively impact on efficient utilisation of outpatient clinics[35]. At the onset of the COVID-19 pandemic, all patients at our institution, including high risk cases with malignant pathologies were assessed virtually without physical presentation to the clinic. Following the easing of restrictions imposed during the pandemic and restoration of conventional physical appointments, telemedicine clinics were exclusively reserved for newly referred patients with benign gallbladder disease and low risk follow up patients.

The concept of telemedicine clinics was introduced long prior to the onset of the COVID-19 pandemic [36, 37]. The obvious benefit to the healthcare provider is a reduction in clinic footfall, with more consultation rooms freed up for other essential clinical contact. Furthermore, the environmental benefit should not be overlooked[38]. In a world where every industry and sector are obligated to consider its carbon footprint, this added bonus is yet to be quantified and may represent future avenues of research. Expectedly, there has been increasing number of reports on the outcomes of telemedicine clinics in surgical practice since the onset of the pandemic.

In a prospective cohort study carried out from September 2021 to March 2022, authors assessed patients’ satisfaction with the telemedicine consultations [39]. Authors concluded that telemedicine assessment is associated with high levels of patient satisfaction regardless of patient age or gender. A small proportion of patients reported that they would prefer an in-person appointment compared to a telemedicine one. However, there is also contrary evidence to suggest that patients would prefer telemedicine follow-up versus in-person, especially for laparoscopic surgery [40]. Further systematic review of randomized controlled trials comparing telemedicine to conventional follow up in general surgery reported similar patient’s satisfaction level to standard care [41].

There have been few randomized controlled trials comparing outcomes of telemedicine and conventional face-to-face follow up appointments for postoperative general surgery patients [36] [4]. Both trials recruited patients for telemedicine postoperative follow up prior to the pandemic onset and included patients undergoing standard routine surgical procedures such as appendicectomy, cholecystectomy and abdominal wall hernia repair. Those studies concluded that telemedicine clinics are a safe and feasible alternative to standard face-to-face follow up for selected patients. Randomized controlled trials have also shown virtual consultations to be safe and effective and to reduce patient-borne costs when measured in a range of surgical and medical specialities including plastic surgery[42], orthopaedic surgery[43] [44] [45] [46], urology [47], chronic kidney disease [48], chronic obstructive pulmonary disease [49] [50], mental health conditions [51] [52], chronic pain [53] [54] [55], adult and teenage diabetes [56] [57] [58].

One study from Mayo clinic assessed video telemedicine clinic in both surgical and non-surgical specialties in terms of concordance of provisional diagnoses established over such clinic visits compared against a reference standard diagnosis which yielded a high degree of diagnostic concordance compared with in person visits for most new clinical concerns[59].

One of the largest retrospective cohort studies assessing telemedicine uptake in surgical practice included 34,875 surgical referrals[60]. The aim of that study was to assess patient and provider characteristics associated with telemedicine uptake including variables such as surgical acuity (benign, urgent, and cancer) and surgical subspecialty, and to determine the predictors for and barriers to telemedicine usage. The benign diagnoses had the largest increase of telemedicine encounters, followed by cancer diagnoses and urgent diagnoses. Authors concluded that surgical specialties were able to shift to telemedicine promptly, safely, and efficiently in the preoperative and postoperative encounters of benign, urgent, and cancer diagnosis during COVID-19 related restrictions.

The main limitations of our study are its retrospective nature, the lack of a control group which would include patients assessed in face-to- face manner, relatively small sample size, no data regarding patients’ and clinicians’ satisfaction. We aimed to review all the cases booked from general surgery telemedicine clinic and operated within the timeframe of the study to ascertain there is no excessive cancellation, complication and readmission rates. The majority of the cases constituted patients with benign gallbladder disease who required day case laparoscopic cholecystectomy.

This study shows that it is feasible and safe to continue utilisation of telemedicine clinics for newly referred patients for certain surgical intervention. We intend to continue utilisation of telemedicine clinic for patients with benign gallbladder pathology which would potentially reduce waiting times and could be more flexible for medical staff and patients. The other advantages for patients would be reduced cost of travel, increased convenience, potentially reduced waiting time for intervention and therefore decreased occurrence of complications whilst waiting for surgery, no need for fixed office space. We advocate to have a low threshold to convert telemedicine clinic into face to face in case there is a doubt about its safety for individual patients or at patient’s request. More work is needed to determine whether or not virtual preoperative assessment is preferential from the patient’s perspective, as well as the fiscal implications of utilising remote consults as more routine practice.

## Data Availability

On request from corresponding author.

## List of abbreviations

NHS: National health service
ASA: American Society of Anaesthesiologists
BMI: Body mass index
